# Editorial: Artificial intelligence for human function and disability

**DOI:** 10.3389/fdgth.2023.1282287

**Published:** 2023-09-08

**Authors:** Denis R. Newman-Griffis, Bart Desmet, Ayah Zirikly, Suzanne Tamang, Chih-Hung Chang

**Affiliations:** ^1^ Information School, University of Sheffield, Sheffield, United Kingdom; ^2^ Rehabilitation Medicine Department, National Institutes of Health Clinical Center, Bethesda, MD, United States; ^3^ Center for Speech and Language Processing, Johns Hopkins University, Baltimore, MD, United States; ^4^ Division of Immunology and Rheumatology, Stanford University School of Medicine, Stanford, CA, United States; ^5^ Program in Occupational Therapy, Washington University School of Medicine, St. Louis, MO, United States; ^6^ Department of Medicine, Washington University School of Medicine, St. Louis, MO, United States; ^7^ Department of Orthopedic Surgery, Washington University School of Medicine, St. Louis, MO, United States

**Keywords:** artificial intelligence, human function, disability, rehabilitation, natural language processing, ICF

**Editorial on the Research Topic** Artificial intelligence for human function and disability

Disability is a near-universal experience, but one for which the transformative potential of health data and artificial intelligence (AI) remains relatively unexplored. The World Health Organization (WHO) estimates that more than one in six people around the world are disabled (1), and many more experience functional limitations associated with frailty, illness, or injury (2). Understanding people's functional outcomes, including impairments to body functions or structures, limitations in activities, and restricted opportunities to participate in society, is vital to capturing the lived experience of health and providing effective, patient-centered care (3, 4). Despite the outstanding global needs for information on function and disability, few studies have explored efficient and cost-effective strategies for bringing informatics and information science to bear on this essential aspect of human health and well-being.

This Research Topic aimed to bring together interdisciplinary perspectives from digital health and health informatics, data science and artificial intelligence, and rehabilitation science to explore the potential of AI technologies in achieving better use of information on human function and disability. AI technologies offer exciting potential for dealing with the significant information challenges posed by function and disability information, such as multiple data sources with diverse data elements and perspectives, data from inside and outside the clinic, and complex, hard-to-standardize data (5). The studies published in this Research Topic represent significant advances in mapping out this potential and charting paths forward for research on AI for function and disability.

The study by Kaelin et al. presents a scoping review of the uses of AI technologies in capturing and analyzing information on participation in the context of pediatric rehabilitation. Participation is notoriously difficult to define and measure, but understanding a patient's participation experiences is key to delivering care that is centered on their unique needs. Their analysis of 21 published studies shows that AI technologies have been explored as part of a variety of assessment methods in pediatric rehabilitation, primarily through *post hoc* analysis of recorded information using machine learning (ML). The measurements analyzed varied widely, including clinical notes, physiological sensors, and face tracking. Their findings highlight clear needs for better reporting on sample characteristics in AI-based rehabilitation studies, the importance of integrating children's perspectives into use of AI in pediatric rehabilitation, the need to explore remote administration of AI-based assessment, and the lack of AI approaches that align with current definitions of participation.

The study by Divita et al. presents new natural language processing (NLP) methods for extracting information on body function from clinical text. Body function has not been systematically studied previously in health NLP research, and presents notable challenges for effective NLP as well as significant value for health professionals. They developed a practical and portable rule-based NLP system for extracting body function information, and tested it on a complex real-world dataset from the U.S. Social Security Administration. Their experiments show promising utility for body function extraction in real-world settings, and their analysis of system performance highlights key challenges for future NLP research on function information to address.

The study by Fan et al. moves outside the clinical setting to analyze consumer product reviews related to chronic pain, a common contributor to—and co-morbidity of—disability. Chronic pain affects more than one in five US adults and can contribute to significant functional limitations and participation restrictions. As a result, many people with chronic pain have developed a range of self-management strategies using over-the-counter and consumer goods, and reviews of these products in online marketplaces can provide an invaluable window into the lived experiences and functioning of people with chronic pain. The study's content analysis shows that online reviews reflect common comorbidities of chronic pain and use of literature-supported, evidence-based strategies for self-management, and further highlight the types of over-the-counter treatment sought and feedback on product efficacy. Their findings provide a valuable characterization of the information available in online reviews for understanding the experience of people with chronic pain, and lays the groundwork for future studies to use online reviews to help identify potential research gaps or therapeutic directions.

Finally, the study by Fu et al. addresses the question of how functional status is documented in current electronic health record systems across a range of healthcare institutions. In increasingly data-driven healthcare, the availability and quality of health information can be a key bottleneck or a major facilitator, particularly for delivering on the promise of learning health systems. Their study draws on the robust, multi-institution population of the Mayo Clinic Study of Aging, and employs a mixed methods approach to identify documentation strategies for functional status across participating healthcare institutions. Their analysis reveals important differences in quality, frequency, and depth of documentation, and illustrates the variation in language, content, and documentation context across different institutions. Their findings provide important insights for the ongoing development of NLP methodologies focused on function and disability, and support their call for ongoing quality assessment of clinical text as a key factor in delivering effective health information.

Together, these studies present valuable insights into the range of health information relevant to human function and disability, and into the role of AI technologies in helping to better collect, analyze, and report function and disability information. They demonstrate the opportunities and challenges in realizing the potential of AI technologies to help analyze and use information on function and disability, reflecting on essential applications of AI in tasks such as information extraction, classification, and prediction. They further illustrate ways in which AI methods, particularly in natural language processing, can help in working with information that is traditionally difficult to collect and standardize, as well as raising awareness of key risks and data quality issues that may limit the benefits of AI use. The work presented in this Research Topic builds on the AI4Function workshops that preceded it (6, 7) to map out important research directions for realizing the benefits of artificial intelligence technologies for human function and disability.
